# Cyclin G2 regulates canonical Wnt signalling via interaction with Dapper1 to attenuate tubulointerstitial fibrosis in diabetic nephropathy

**DOI:** 10.1111/jcmm.14946

**Published:** 2020-01-24

**Authors:** Chenyang Zhao, Jinlan Gao, Sen Li, Qi Liu, Xiaoyu Hou, Xuesha Xing, Danning Wang, Manni Sun, Shusen Wang, Yang Luo

**Affiliations:** ^1^ The Research Center for Medical Genomics Key Laboratory of Cell Biology Ministry of Public Health Key Laboratory of Medical Cell Biology Ministry of Education School of Life Sciences China Medical University Shenyang China

**Keywords:** cyclin G2, Dapper1, diabetic nephropathy, tubulointerstitial fibrosis, Wnt signalling

## Abstract

Cyclin G2 (*CCNG2*) is an atypical cyclin that inhibits cell cycle progression and is often dysregulated in human cancers. Cyclin G2 in the occurrence and development of diabetic nephropathy (DN), one of the most severe diabetic complications, has not been fully identified. In this study, we investigated the function and regulatory mechanism of cyclin G2 in DN. In vivo studies revealed that a deficiency of cyclin G2 significantly increased albuminuria and promoted tubulointerstitial fibrosis in established DN. Cyclin G2 regulated the expression of fibrosis‐related proteins via the canonical Wnt signalling pathway in renal tubular epithelial cells. Moreover, the binding of cyclin G2 to Dapper1 (Dpr1/DACT1), a protein involved in Wnt signalling, decreased the phosphorylation of Dpr1 at Ser762 by casein kinase 1 (CK1) and suppressed the Wnt signalling pathway. These findings reveal that cyclin G2 can protect against renal injury and fibrosis associated with DN and, thus, is a new target for the prevention and treatment of diabetic complications.

## 1 INTRODUCTION

Cyclin G2 (*CCNG2*) is a noncanonical cyclin that belongs to the same family as cyclin I and cyclin G1. Unlike other cyclins, the expression of cyclin G2 increases in cycle‐arrested and terminally differentiated cells.^1^, ^2^ A large body of evidence indicates that cyclin G2 acts as an important tumour suppressor.^3^, ^4^, ^5^, ^6^ Recent findings have suggested that the expression of cyclin G2 in fatty tissues of obese patients is associated with steady‐state of carbohydrate metabolism in the body.^7^ Furthermore, in patients with type 2 diabetes, the level of cyclin G2 is positively correlated with that of insulin‐degrading enzyme (IDE).^7^ Moreover, insulin and insulin‐like growth factor 1 (IGF‐1) can significantly down‐regulate the expression of cyclin G2, thus stimulating DNA synthesis and promoting cell proliferation.^8^ In addition, our recent study found that cyclin G2 expression plays an important role in controlling glioma progression by regulating proliferation and the Warburg effect via its interaction with lactate dyhydrogenase A (LDHA).^9^ Additionally, other studies have provided unbiased evidence that the Warburg effect plays a pivotal role in the development of diabetes.^10^, ^11^ These lines of evidence suggest that cyclin G2 may be involved in the pathological processes related to diabetes.

Diabetic nephropathy (DN) is a relatively common chronic microvascular complication of diabetes and the primary cause of end‐stage renal disease.^12^, ^13^, ^14^ The pathological features of DN include renal glomerular hypertrophy, thickening of the glomerular and tubular basement membrane, extracellular matrix accumulation, and eventual tubular interstitial fibrosis and glomerulosclerosis. The clinical outcome of DN involves progressive, irreversible renal dysfunction and ultimately renal failure.^15^, ^16^ The pathogenesis of DN is not fully understood, and effective approaches for the treatment and prevention of DN are lacking. Therefore, studies that address the molecular pathogenesis of DN are vital. Previously, we reported that cyclin G2 attenuated glomerulosclerosis in DN through the Wnt pathway.^17^ However, it has recently been demonstrated that the development of tubulointerstitial lesions is more closely correlated with a progressive decline in renal function, compared with glomerular lesions,^16^, ^18^, ^19^, ^20^, ^21^, ^22^ and the function of cyclin G2 in tubulointerstitial fibrosis is not fully identified.

The canonical Wnt signalling pathway is an evolutionarily conserved, developmental signalling system that plays an important role in organ development and tissue homeostasis. The Wnt signalling pathway is activated in various kidney diseases such as obstructive nephropathy, diabetic nephropathy and polycystic kidney disease.^23^, ^24^, ^25^, ^26^ The downstream proteins associated with Wnt signalling are up‐regulated in the kidneys of patients with diabetes and in diabetic mouse models.^27^, ^28^ Similarly, high‐glucose induction activates the Wnt signalling pathway in glomerular podocytes and mesangial cells and causes excessive apoptosis of intrinsic renal cells.^27^, ^29^ Suppression of Wnt signalling improves proteinuria and tubulointerstitial fibrosis in patients with type 1 diabetes.^30^ As a central mediator of Wnt signalling, Dishevelled (Dvl) plays an important role in both β‐catenin–mediated canonical and β‐catenin–independent noncanonical Wnt signalling.^31^ Dpr1, originally identified as a Dvl‐interacting protein,^32^ has been shown to mediate Dvl degradation and thus inhibit both Dvl‐mediated canonical and noncanonical Wnt signalling.^33^, ^34^, ^35^

In this study, we investigated the involvement of cyclin G2 in pathological processes associated with DN, both in vitro and in vivo*,* using several molecular biology techniques. We found that cyclin G2 attenuates the development of tubulointerstitial fibrosis in DN by regulating Wnt signalling via interaction with Dpr1. Our results highlight a novel role for cyclin G2 as a mediator of metabolic disease progression.

## 2 MATERIALS AND METHODS

### 2.1 Mouse model of streptozotocin‐induced diabetes

Whole‐body cyclin G2 knockout C57BL/6 mice (*Ccng2^−/−^*) were generated at the Shanghai Model Organisms Center (China). Male wild‐type (WT; C57BL/6, n = 25) and *Ccng2^−/−^* mice (n = 27) were housed in cages, fed standard chow and maintained on a 12‐hour light‐dark cycle. Diabetes was induced as described previously.^36^ Briefly, 8‐week‐old mice received five consecutive intravenous injections of streptozotocin (STZ) (50 mg/kg; Sigma‐Aldrich; V900890) in citrate buffer (pH 4.6) or citrate buffer only. A blood glucose level >11.2 mmol/L was confirmed 3 days after STZ administration at three different time‐points. Mice with blood glucose levels less than 11.2 mmol/L at 2 weeks after the final injection of STZ were excluded from the experiment. Metabolic cages were used for collection of urine over a 24‐hour period. Urinary albumin levels were measured using the QuantiChrom BCG Albumin Assay Kit (BioAssay Systems; DIAG‐250); urinary creatinine levels were measured using the Parameter Creatinine Assay (R&D Systems; KGE005). Mice were killed 16 weeks after STZ injection, and an age‐matched WT (n = 6) or *Ccng2^−/−^* mouse (n = 6) was also killed at the same time (Figure S1). All institutional and national guidelines for the care and use of laboratory animals were followed. All animal experiments were approved by the Animal Care and Use Committee of the Department of Animal Resources, China Medical University.

### 2.2 Morphological studies

Renal tissue specimens were fixed in 4% paraformaldehyde and embedded in paraffin. Sections of 4‐μm thickness were examined by haematoxylin‐eosin (HE) staining and Masson's trichrome staining (Solarbio life sciences; G1120; G1340) as well as immunohistochemistry assays.

The percentage fibrosis area was determined from 15 fields of Masson's trichrome‐stained specimens viewed at 200× magnification. Lesions were quantified using Image‐Pro Plus software. The fibrotic area was digitized and subjected to colour‐threshold analysis. Scores from ten non‐overlapping fields per kidney were averaged to obtain the final percentage fibrosis area.

For immunohistochemistry, sections were deparaffinized, rehydrated and autoclaved for 10 minutes in citrate buffer for antigen retrieval. Nonspecific binding was blocked by incubation with 10% goat or rabbit serum for 30 minutes. The samples were incubated with anti–β‐catenin (Sigma‐Aldrich; C2206), anti‐collagen IV (Abcam; ab6586) or anti–N‐cadherin (Cell Signaling Technology; 13116) primary antibodies at 4°C overnight. After washing in PBS, the sections were incubated with an appropriate secondary antibody and detected using the Ultrasensitive S‐P Kit (streptavidin‐peroxidase; Sigma‐Aldrich; S2438).

### 2.3 Cell culture, transfection and treatment

Cells from a human renal tubular epithelial cell line (HK‐2) were purchased from the ATCC and cultured with DMEM containing 10% foetal bovine serum (Gibco, Life Technologies). Cells were infected with lentivirus particles harbouring *CCNG2* or control GFP lentivirus (GeneChem) and divided into high‐glucose (HG; 30 mmol/L d‐glucose) and low‐glucose (LG; 5.5 mmol/L d‐glucose and 24.5 mmol/L l‐glucose) treatment groups (Figure S2).^37^, ^38^ Cells were starved by incubation in DMEM containing 1% serum for 24 hours, and then, the media was replaced with DMEM containing 10% serum and the indicated concentrations of glucose. Cells were infected with lentivirus particles harbouring *CCNG2* and exposed to the Wnt signalling activator CHIR99021 (1 mmol/L; R&D Systems; 4423) or an equivalent volume of DMSO in DMEM (negative control) for 72 hours.

The eukaryotic expression vectors pCMV‐3 × FLAG‐G2, pEGFP‐CK1, pCMV‐Tag5A‐Dpr1 and PGPU6/GFP/Neo‐shDACT1 were prepared. HK‐2 cells were cultured to 80% confluency, then transfected with one or more of the following recombinant expression vectors: p3 × FLAG‐CMV‐BAP (Sigma‐Aldrich), pCMV‐3 × FLAG‐G2, pEGFP‐N3, PGPU6/GFP/Neo (BD Biosciences), pEGFP‐CK1, PGPU6/GFP/Neo‐shDACT1, pCMV‐Tag5A (Huayueyang Bio), pCMV‐Tag5A‐Dpr1 or pCMV‐Tag5A‐Dpr1 S762A. Transfections were performed using Lipofectamine 2000 (Life Technologies; 11668500) according to the manufacturer's instructions.

### 2.4 Immunoprecipitation and Western blot analysis

Total cellular protein (3 mg) from HK‐2 cells were incubated with an anti‐Dpr1 antibody (Abcam; ab51260) and Protein G Plus Agarose (Santa Cruz Biotechnology; sc‐500778) at 4°C overnight. After washing, Ser/Thr‐phosphorylated proteins were isolated using a phosphorylation purification kit according to the manufacturer's instructions (Qiagen, Life Technologies; 37145). The purified protein concentration was adjusted to 0.1 mg/mL; a 30‐µL aliquot was used for Western blotting. Western blotting was performed using an anti‐phosphoserine/threonine antibody (Sigma‐Aldrich; P3430).

For Western blotting, protein concentrations were determined using BCA (Life Technologies; 23227). The anti–E‐cadherin (#3195), anti–N‐cadherin (#13116), anti‐cyclin D1 (#2978), anti–β‐tubulin (#86298), anti–phospho‐β‐catenin (Ser33/37/Thr41) (#9561) and anti‐Dvl2 (#3224) antibodies were from Cell Signaling Technology. The anti‐collagen IV (ab6586) antibody was from Abcam. The anti‐GSK3β (sc‐71186) and anti–phospho‐GSK3β (Ser9) (sc‐11757) antibodies were from Santa Cruz Biotechnology. The anti–β‐catenin (C2206) and anti‐cyclin G2 (HPA034684) antibodies were from Sigma‐Aldrich, and the anti‐MMP7 (10374‐2‐AP) antibody was from Proteintech. All experiments were performed in triplicate.

### 2.5 In situ proximity ligation assay

For in situ proximity ligation assays (PLA) (Duolink; Sigma‐Aldrich; DUO92103), oligonucleotide‐conjugated ‘PLA probe’ antibodies were directed against the primary antibodies for Dpr1, cyclin G2 or CK1. The annealing of the PLA probes occurred when Dpr1, cyclin G2 or CK1 was in close proximity, which initiated the amplification of repeat sequences recognized by the fluorescently labelled oligonucleotide probe. Briefly, HK‐2 cells were seeded on glass‐bottom cell culture dishes (Nest Biotechnology) and stimulated as described.^39^, ^40^ The cells were washed with ice‐cold phosphate‐buffered saline (PBS) and fixed with 4% paraformaldehyde for 20 minutes at room temperature. The cells were permeabilized with 0.1% Triton X‐100 for 20 minutes at room temperature. The primary antibodies used in this study were anti‐Dpr1 (Abcam; ab51260; Santa Cruz Biotechnology; sc‐74599), anti‐cyclin G2 (Sigma‐Aldrich; HPA034684) and anti‐CK1 (Santa Cruz Biotechnology; sc‐6478). The PLA probes were anti‐goat (minus) and anti‐rabbit (plus). Duolink in situ Detection Reagent Red was used for detection. Imaging was performed using a confocal fluorescence microscope. Images were analysed with Image‐Pro Plus software. At least 50 cells were analysed in each experiment.

### 2.6 Immunofluorescence

Cells were fixed, permeabilized and then blocked with bovine serum albumin (BSA) for 30 minutes. The cells were incubated with primary antibody at 4°C overnight and then incubated with secondary antibody for 45 minutes at room temperature. DAPI was used to stain the nuclei. Imaging was performed using an inverted fluorescence microscope.

### 2.7 Statistical analysis

Data were expressed as the mean ± standard deviation (SD). Statistical analyses were performed using a Student's *t* test for comparisons between two groups or using a one‐way ANOVA for analyses between three or more groups. Statistical significance was defined as *P* < .05.

## 3 RESULTS

### 3.1 Cyclin G2 expression is abnormally decreased in kidneys of DN mice and high glucose–induced renal tubular epithelial cells

**Figure 1:**
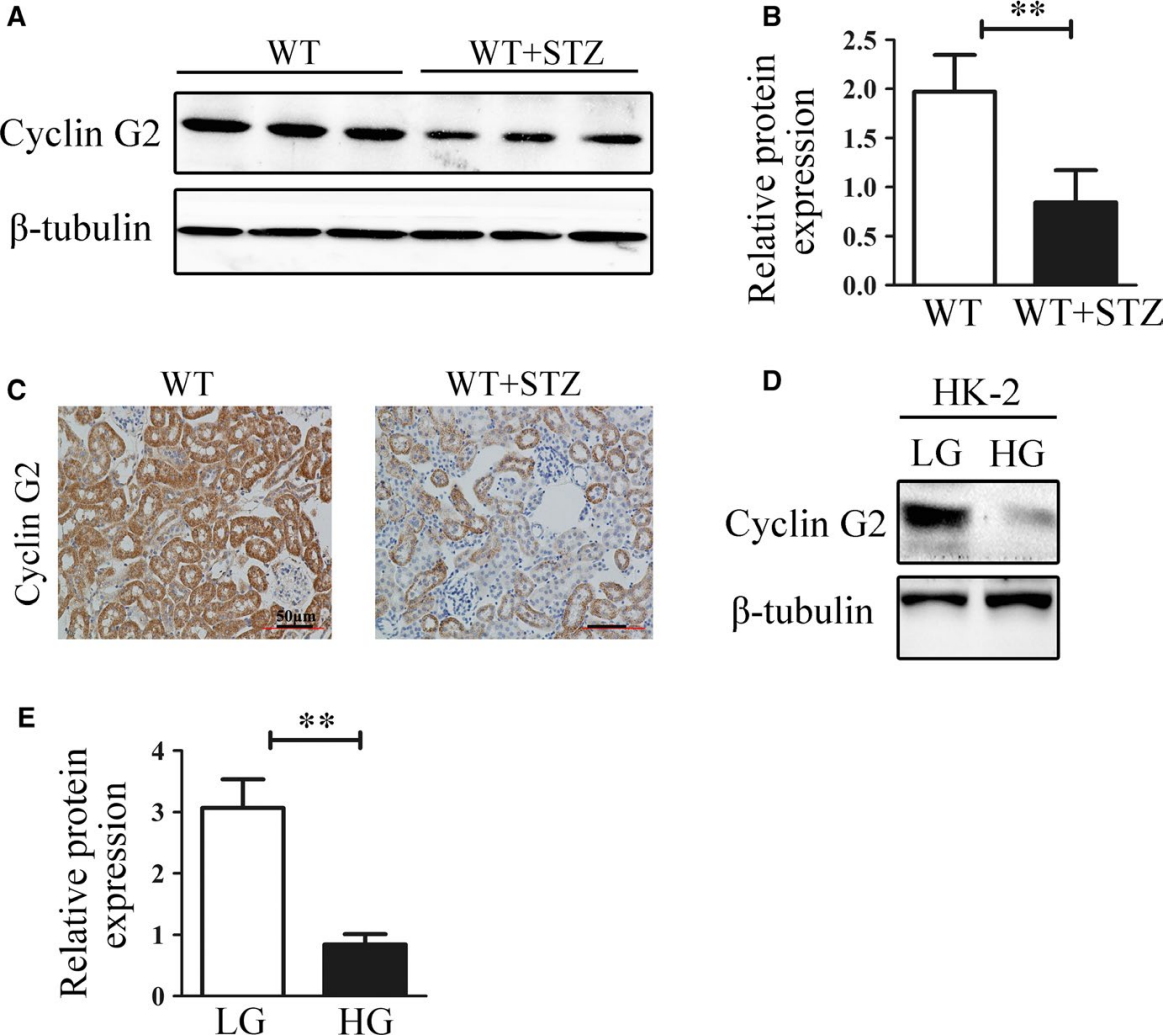
Down‐regulation of cyclin G2 expression in DN mice and high glucose–induced HK‐2 cells. A, B, Representative Western blots and densitometry results show cyclin G2 protein levels in renal tissue homogenate of normal mice and STZ‐induced DN mice. C, Immunohistochemical staining (brown) show cyclin G2 protein levels in renal tissues of normal mice and STZ‐induced DN mice (original magnification, ×200, n = 6). D, E, Representative Western blots and densitometry results show cyclin G2 protein levels in low and high glucose–induced HK‐2 cells. (n = 3). Values are expressed as the mean ± SD; *P < .05; **P < .01; *n.s.*, not significant. Three independent experiments were performed

Cyclin G2 expression was evaluated by Western blotting using the renal tissue homogenate of normal mice and those with STZ‐induced DN at 16 weeks after the onset of diabetes. The protein expression of cyclin G2 was significantly decreased in the renal tissue homogenate of DN mice (Figure 1A,B). Immunohistochemical staining showed that the protein expression of cyclin G2 was down‐regulated in renal tissues from STZ‐induced DN mice (Figure 1C). We further examined cyclin G2 protein expression in HK‐2 cells cultured with low‐ and high‐glucose concentrations. The protein expression level of cyclin G2 was significantly decreased in HK‐2 cells cultured with high glucose (Figure 1D,E).

### 3.2 Cyclin G2 deficiency increases albuminuria in DN mice

**Figure 2:**
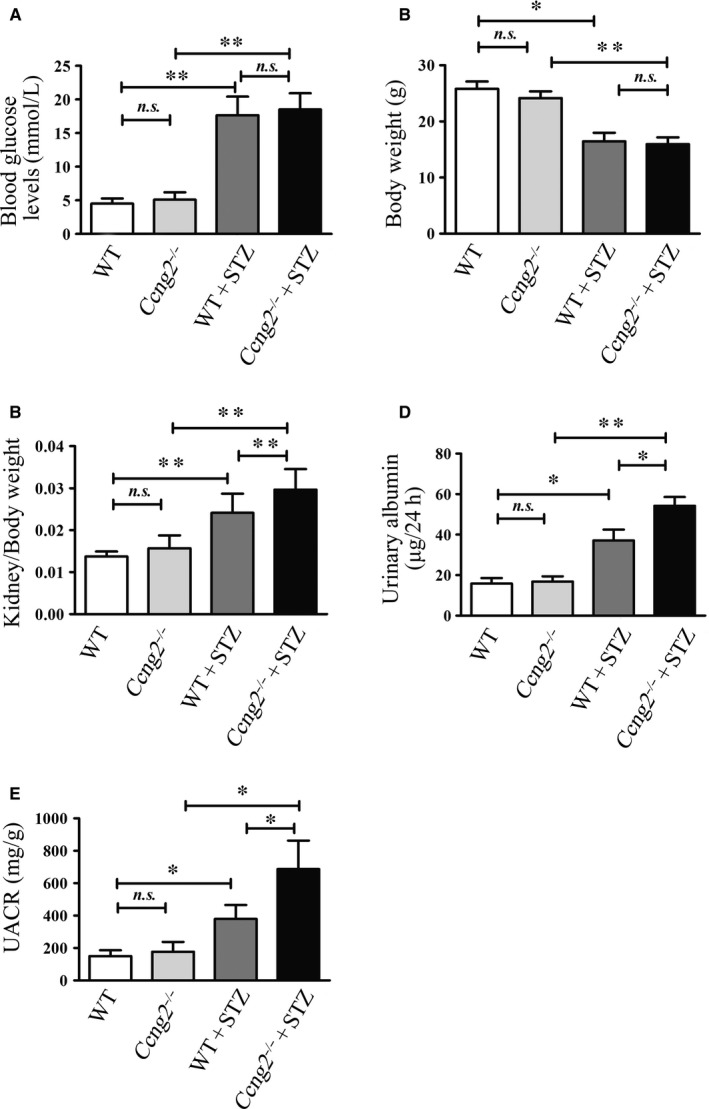
A deficiency in cyclin G2 increased proteinuria in a DN mouse model. A‐E, *Ccng2^−/−^* and WT mice were treated with citrate buffer or STZ. Blood glucose levels (mmol/L), body weight (g), kidney/body weight, urinary albumin (μg/24 h) and urinary albumin/creatinine ratio (mg/g) were determined (n = 6). Values are expressed as the mean ± SD; *P < .05; **P < .01; *n.s.*, not significant. Three independent experiments were performed

To evaluate whether cyclin G2 protects against renal dysfunction developed in diabetes, we assessed blood glucose, 24‐hour urinary albumin, urinary creatinine, body weight and kidney weight in our animal model. Blood glucose, renal weight ratio, urinary albumin and urinary albumin/creatinine ratio (UACR) were unchanged in *Ccng2^−/−^* mice as compared to WT control mice in the absence of STZ treatment. However, the 24‐hour urinary albumin levels and the UACR were significantly increased in the *Ccng2^−/−^* mice following the induction of DN by STZ as compared with DN WT mice. The renal weight ratio was also higher in the DN *Ccng2^−/−^* group, but blood glucose levels were not significantly increased (Figure 2A‐E). Therefore, a lack of cyclin G2 may cause an increase in proteinuria in DN mice.

### 3.3 Cyclin G2 deficiency increases the severity of renal injury and tubulointerstitial fibrosis in DN mice

**Figure 3:**
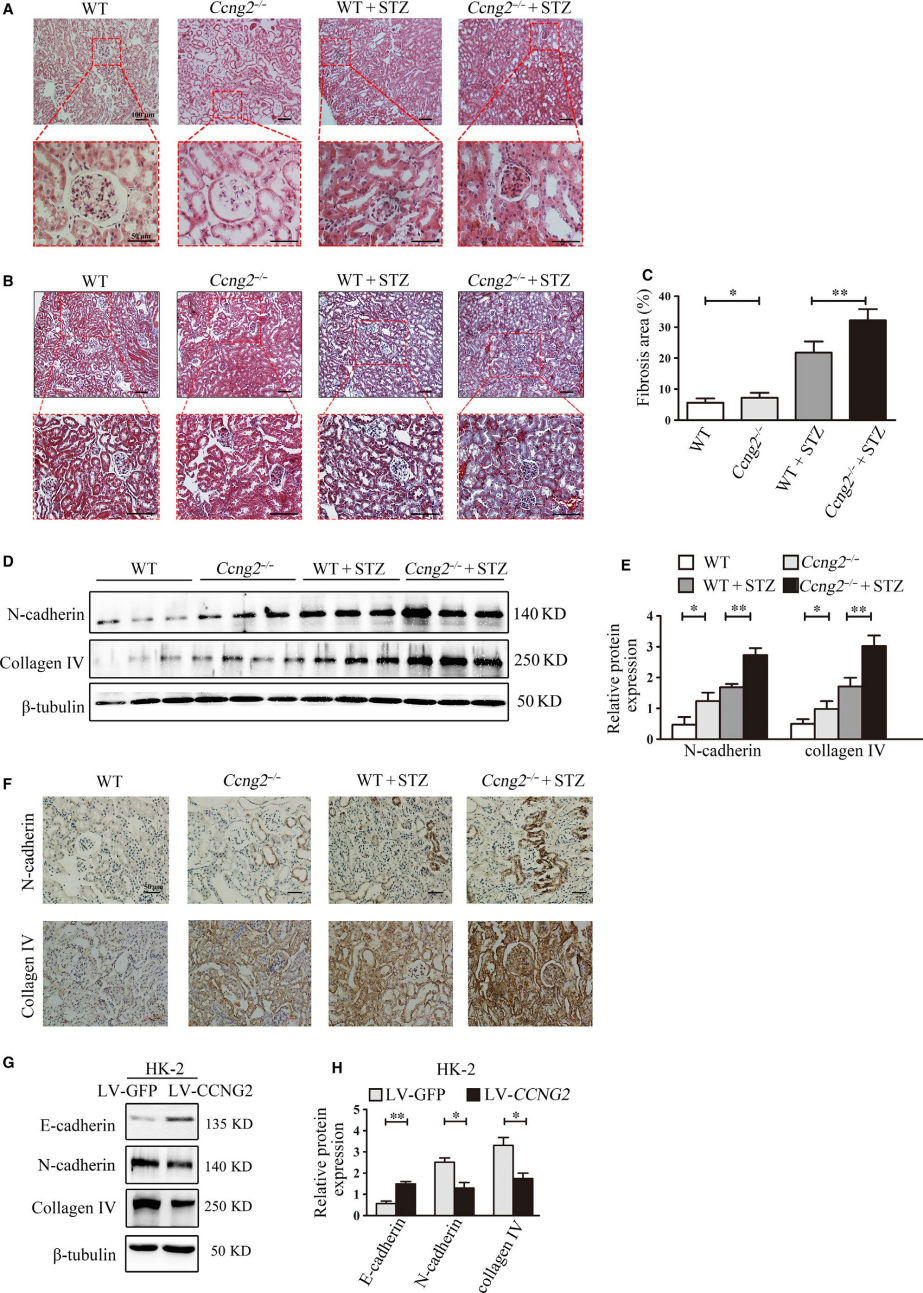
A deficiency in cyclin G2 contributes to renal injury and fibrosis in DN mice. A, HE staining identified pathological renal alterations in WT mice, *Ccng2^−/−^* mice, STZ‐induced DN WT mice and STZ‐induced DN *Ccng2^−/−^* mice (original magnification, 100×, 400×, n = 6). B, C, Masson's trichrome staining identified an interstitial fibrosis area (original magnification, 100×, 200×, n = 6). D, Western blot and (E) densitometry analyses of N‐cadherin and collagen IV in the renal cortex in WT mice, *Ccng2^−/−^* mice, STZ‐induced DN WT mice and STZ‐induced DN *Ccng2^−/−^* mice (n = 6). F, Representative immunohistochemistry of the expression of N‐cadherin and collagen IV in WT mice, *Ccng2^−/−^* mice, STZ‐induced DN WT mice and STZ‐induced DN *Ccng2^−/−^* mice (original magnification, 200×, n = 6). G, H, Western blotting and densitometry for E‐cadherin, N‐cadherin and collagen IV in HK‐2 cells (n = 3). The values are expressed as the mean ± SD; *P < .05; **P < .01; *n.s.*, not significant. Three independent experiments were performed

Pathological alterations in the renal tissues of DN mice were determined by HE staining (Figure 3A). Masson's trichrome staining was used to evaluate the collagen fibres in the renal cortex. Cyclin G2 deletion in mice resulted in pathological changes, including renal tubular atrophy, tubular dilation and interstitial fibrosis. In addition, the largest areas of fibrosis were observed in the renal tissues of DN *Ccng2^−/−^* mice (Figure 3B,C).

To evaluate the contribution of cyclin G2 on fibrosis‐related proteins in vivo, we determined the protein levels of N‐cadherin and collagen IV in the renal tissues from *Ccng2^−/−^* and WT mice following the induction of DN. Western blotting and immunohistochemistry revealed that DN *Ccng2^−/−^* mice had larger increases in N‐cadherin and collagen IV levels as compared to DN WT mice (Figure 3D‐F). Hence, cyclin G2 might attenuate tubulointerstitial fibrosis and relieve renal injury in this animal model of diabetes by decreasing the changes in fibrosis‐related proteins induced during the development of DN.

In parallel, we evaluated whether cyclin G2 could abrogate tubulointerstitial fibrosis in vitro by overexpressing cyclin G2 in HK‐2 cells using lentiviral particles (Figure 3G,H) and measuring N‐cadherin, E‐cadherin and collagen IV protein levels under conditions of high glucose. At normal cyclin G2 levels, N‐cadherin and collagen IV were up‐regulated in HK‐2 cells under high‐glucose conditions compared to the low‐glucose control group; by contrast, E‐cadherin was down‐regulated. Overexpression of cyclin G2 suppressed high glucose–induced up‐regulation of N‐cadherin and collagen IV (Figure S3).

### 3.4 Cyclin G2 down‐regulates the expression of tubular fibrosis‐related proteins by regulating canonical Wnt signalling

**Figure 4:**
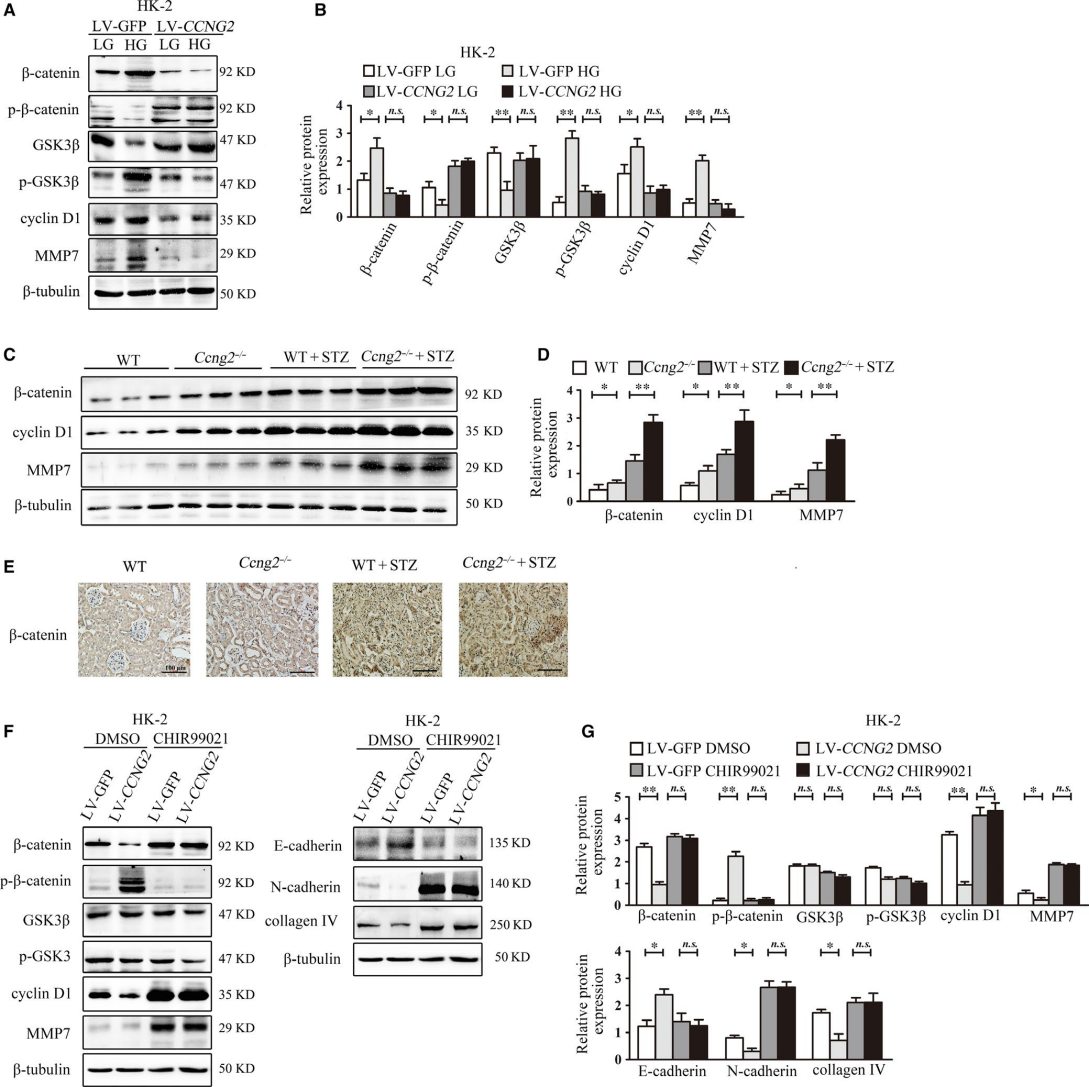
Cyclin G2 inhibits the expression of proteins associated with tubulointerstitial fibrosis via canonical Wnt signalling. A, B, Western blotting and densitometry for β‐catenin, GSK3β, cyclin D1 and MMP7 in *CCNG2*‐overexpressing HK‐2 cells exposed to 30 mmol/L D‐glucose (HG) or 5.5 mmol/L D‐glucose + 24.5 mmol/L l‐glucose (LG; control group) for 72 h (n = 3). C, D, Western blotting and densitometry results of β‐catenin, cyclin D1 and MMP7 in the renal cortex of WT mice, *Ccng2^−/−^* mice, STZ‐induced DN WT mice and STZ‐induced DN *Ccng2^−/−^* mice (n = 6). E, Representative immunohistochemistry of the expression of β‐catenin in WT mice, *Ccng2^−/−^* mice, STZ‐induced DN WT mice and STZ‐induced DN *Ccng2^−/−^* mice (original magnification, 200×, n = 6). F, G, Western blotting and densitometry of β‐catenin, p‐β‐catenin, GSK3β, p‐GSK3β, cyclin D1, MMP7, E‐cadherin, FN, N‐cadherin and collagen IV in cyclin G2–overexpressing HK‐2 cells (LV‐*CCNG2*) treated with 5 μmol/L CHIR99021 or DMSO (control group) for 72 h (n = 3). Values are expressed as the mean ± SD; *P < .05; **P < .01; *n.s.*, not significant. Three independent experiments were performed

Other researchers have noted that activation of Wnt signalling induces renal injury and fibrosis.^16^, ^41^, ^42^ Therefore, we investigated whether cyclin G2 could regulate the progression of tubulointerstitial fibrosis through Wnt signalling. Specifically, we assessed the effect of cyclin G2 on the protein expression of Wnt signalling factors in vitro and in vivo. As expected, ectopic expression of cyclin G2 inhibited the expression of β‐catenin (ie the key regulator of the Wnt signalling pathway), p‐GSK3β and its targets (cyclin D1 and MMP7); meanwhile p‐β‐catenin and GSK3β were up‐regulated in high glucose–induced HK‐2 cells (Figure 4A,B). In addition, the levels of β‐catenin, cyclin D1 and MMP7 in DN *Ccng2^−/−^* mice were substantially higher than those of DN WT mice (Figure 4C‐E). These results suggested that cyclin G2 negatively regulates the Wnt pathway in high glucose–induced HK‐2 cells as well as in mouse kidney.

To discover the mechanisms by which cyclin G2 influences tubulointerstitial fibrosis, we activated the Wnt signalling pathway using the GSK3β inhibitor CHIR99021.^43^, ^44^ Before CHIR99021 addition, cyclin G2 overexpression inhibited both the expression of proteins associated with Wnt signalling and tubular interstitial fibrosis. After the addition of CHIR99021, the inhibition of Wnt signalling factor expression was abolished. Moreover, down‐regulation of N‐cadherin and collagen IV and up‐regulation of E‐cadherin were diminished (Figure 4F,G). These data indicate that cyclin G2 can regulate the expression of tubular interstitial fibrosis‐related proteins via Wnt signalling in HK‐2 cells.

### 3.5 Cyclin G2 interacts with Dpr1 and decreases CK1‐mediated phosphorylation of Dpr1

**Figure 5:**
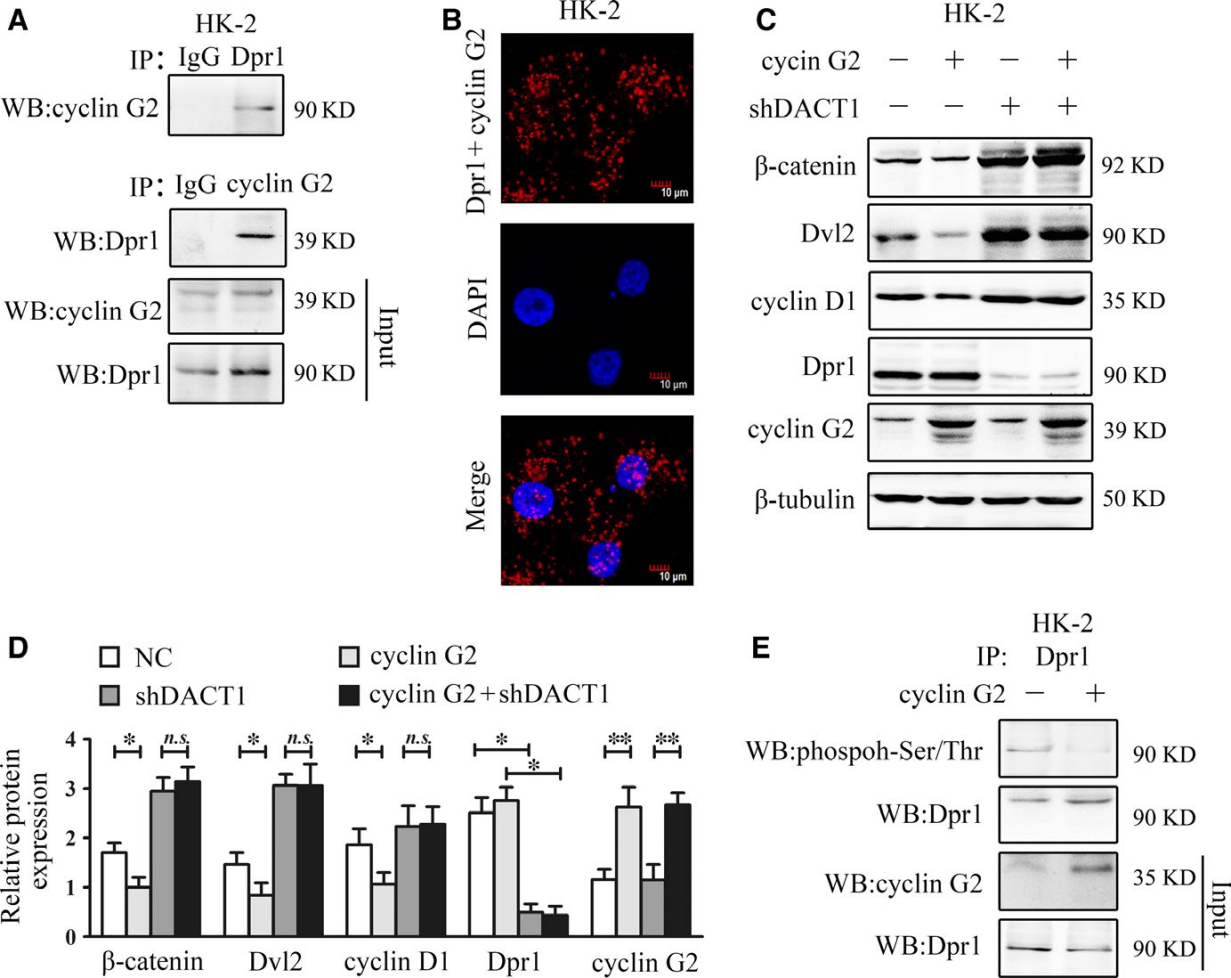
Cyclin G2 decreases the phosphorylation of Dpr1. A, B, The interaction of Dpr1 with cyclin G2 was examined by coimmunoprecipitation and Duolink in situ PLA. Red, sites of interaction of Dpr1 with cyclin G2; blue, nuclear staining (original magnification, 600×, n = 3). C, D, HK‐2 cells were transfected with PGPU6/GFP/Neo‐shDACT1 and pCMV‐3 × FLAG‐G2 or control plasmids for 48 h followed by the detection of Dvl2, β‐catenin and cyclin D1 (n = 3). E, HK‐2 cells were transfected with pCMV‐3 × FLAG‐G2 or control plasmid (p3 × FLAG‐CMV‐BAP) for 48 h. Lysates were immunoprecipitated with anti‐Dpr1 antibody and immunoblotted with anti‐phosphoserine/threonine antibody (n = 3). Values are expressed as the mean ± SD; *P < .05; **P < .01; *n.s.*, not significant. Three independent experiments were performed

To explore the mechanisms by which cyclin G2 regulates Wnt signalling, we conducted yeast two‐hybrid experiments and screened cyclin G2‐conjugated proteins, which identified Dpr1, a Dvl binding antagonist protein.^45^ Coimmunoprecipitation and Duolink in situ proximity ligation assays (PLA) were used to confirm that Dpr1 interacted with cyclin G2 in HK‐2 cells (Figure 5A,B). Silencing of Dpr1 induced the expression of Dvl2, β‐catenin and cyclin D1. In contrast, cyclin G2 inhibited the expression of these three proteins. However, the inhibition of Dvl2, β‐catenin and cyclin D1 expression by cyclin G2 was blocked after Dpr1 silencing (Figure 5C,D). These results indicate that cyclin G2 suppresses Dvl2 expression and up‐regulates GSK3β activity by binding to Dpr1 and suppressing the Wnt signalling pathway.

Other authors have reported that the phosphorylation level of Dpr1 determines whether Wnt signalling is inhibited or activated.^46^ Therefore, we investigated whether cyclin G2 affected the phosphorylation level of Dpr1. Because no reference site or antibody for Dpr1 phosphorylation had been described previously, we determined the level of Dpr1 phosphorylation using an anti‐phosphoserine/threonine antibody after coimmunoprecipitation with an anti‐Dpr1 antibody. Overexpression of cyclin G2 in HK‐2 cells reduced the level of phosphorylated Dpr1 (Figure 5E).

**Figure 6:**
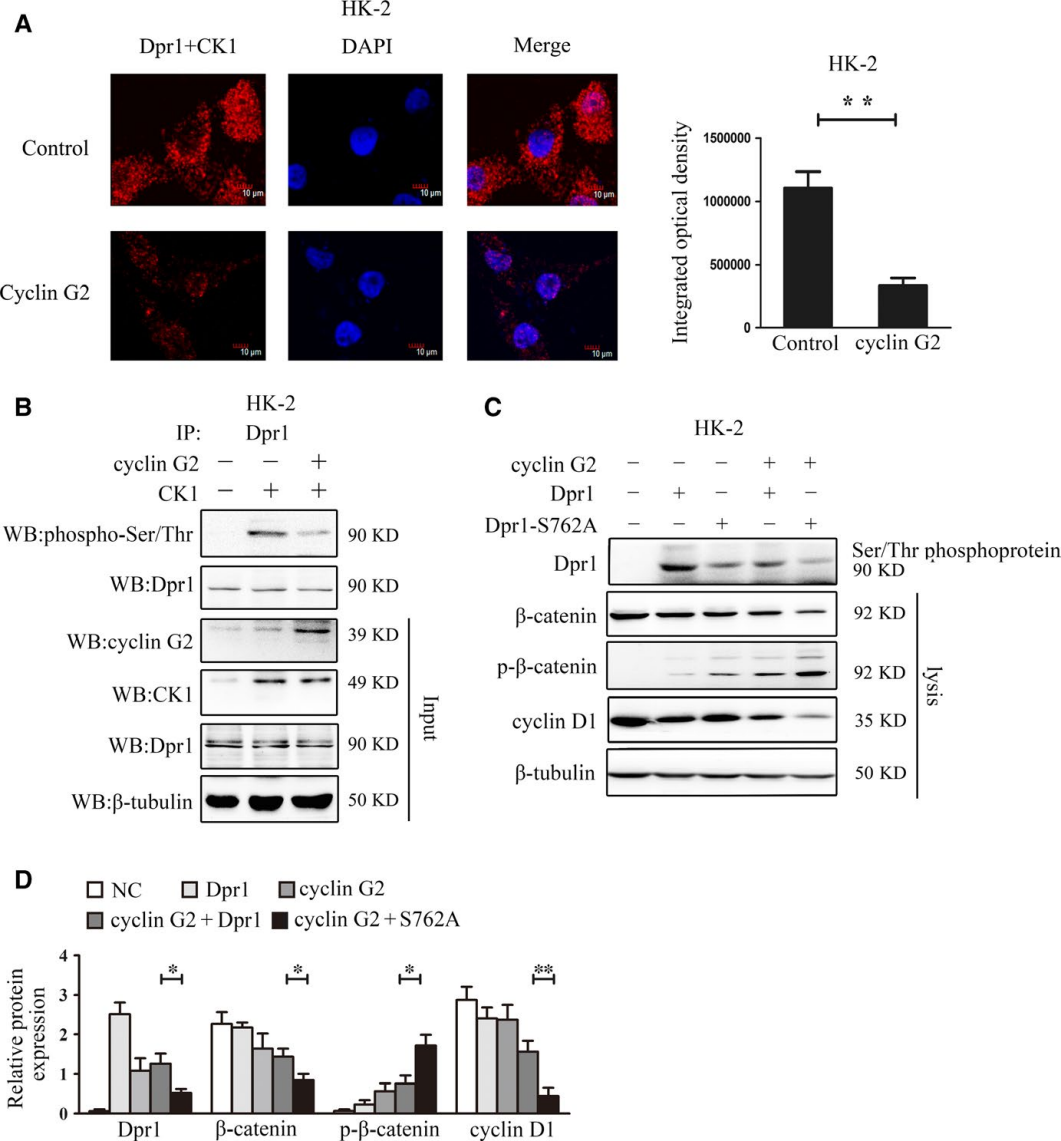
Cyclin G2 decreases Ser762 phosphorylation of Dpr1 by CK1. A, The interaction of Dpr1 with CK1 was detected using Duolink in situ PLA in the context of cyclin G2 overexpression. Red, sites of interaction of Dpr1 with CK1; blue, nuclear staining (original magnification, 600×, n = 3). B, HK‐2 cells were transfected with pCMV‐3 × FLAG‐G2 or pCMV‐3 × FLAG‐G2 and pEGFP‐CK1 for 48 h. Lysates were immunoprecipitated with anti‐Dpr1 antibody and immunoblotted with anti‐phosphoserine/threonine antibody (n = 3). C, D, HK‐2 cells were transfected with pCMV‐3 × FLAG‐G2, pCMV‐Tag5A‐Dpr1 and pCMV‐Tag5A‐Dpr1 S762A for 48 h followed by the detection of β‐catenin, p‐β‐catenin and cyclin D1 (n = 3). Values are expressed as the mean ± SD; *P < .05; **P < .01; *n.s.*, not significant. Three independent experiments were performed

It was previously shown that Dpr1 is unable to bind to Dvl2 and inhibit Wnt signalling following its phosphorylation by CK1.^46^ We presumed that the binding of cyclin G2 could affect Dpr1 phosphorylation by CK1. Using a Duolink in situ PLA, we detected a reduction in the binding between Dpr1 and CK1 after overexpression of cyclin G2 in HK‐2 cells (Figure 6A). In contrast, overexpression of CK1 up‐regulated the phosphorylation level of Dpr1. However, co‐overexpression of CK1 and cyclin G2 decreased the phosphorylation level of Dpr1 (Figure 6B). These findings reveal that cyclin G2 interacts with Dpr1 and inhibits its phosphorylation by CK1, thus negatively regulating the Wnt signalling pathway.

### 3.6 Cyclin G2 decreased the phosphorylation of Dpr1 Ser762 site

Using KinasePhos and Scansite software,^47^, ^48^ we predicted the effects of cyclin G2 on the phosphorylation of Dpr1 by CK1 (Figure S4). The results predicted that Ser762 was the CK1 phosphorylation site on Dpr1. To investigate this possibility, amino acid 762 located in the Dpr1 C‐terminus was point‐mutated to alanine (S762A). Ser/Thr‐phosphorylated proteins were collected, and the effects of cyclin G2 on Dpr1 phosphorylation were analysed. Overexpression of cyclin G2 more significantly decreased the phosphorylation level of mutant Dpr1 as compared with that of the wild‐type Dpr1. Furthermore, the levels of phosphorylated β‐catenin were decreased in cells transfected with wild‐type Dpr1; this effect was abolished in the S762A‐mutant Dpr1 (Figure 6C,D). These findings suggest that cyclin G2 plays a role in the Ser762 phosphorylation of Dpr1 by CK1.

## 4 DISCUSSION

The pathogenesis of DN is a complex process involving multiple factors and many molecular mechanisms. Metabolic factors and activation of some signalling pathways are thought to be the driving force behind renal cell injury and DN.^49^, ^50^ Cyclin G2 is an unconventional cyclin that can function as a positive regulator of adipocyte differentiation through the transcriptional activity of PPARγ.^51^ Recently, we reported that cyclin G2 expression plays an important role in controlling glioma progression by regulating proliferation and the Warburg effect,^9^ which play pivotal roles in the development of DN. These results suggest that cyclin G2 likely plays additional roles in cell signalling and metabolic processes. Our previous research has demonstrated that cyclin G2 has a role in inhibiting glomerulosclerosis in DN,^17^ but increasing evidence showed that the degree of renal function injury is more closely related to tubulointerstitial fibrosis.^22^ In this study, we found low expression of cyclin G2 in the kidneys of STZ‐induced diabetic mice and high glucose–induced HK‐2 cells (Figure 1), suggesting that cyclin G2 could be involved in modulating tubulointerstitial fibrosis in DN; the mechanism by which this may occur requires further exploration.

Recent reports consistently point to cyclin G2 as having cell cycle inhibitory functions.^52^, ^53^ Cells with cyclin G2 deficiency are more likely to evolve into malignant tumours. Our results have shown that cyclin G2 is down‐regulated in DN mice. Taken together, these results led us to consider the possibility of cyclin G2 deficiency contributes to renal cell injury. In our study, cyclin G2 deficiency increased the severity renal injury and tubulointerstitial fibrosis in DN mice compared with STZ‐induced DN WT mice. In addition, collagen fibres were markedly increased and tubulointerstitial extracellular matrix accumulated in DN *Ccng2^−/−^* mice. Furthermore, we found that fibrosis‐related proteins were also elevated and other pathophysiological changes were evident (Figure 3). These results suggest a protective function for cyclin G2 in diabetic kidney complications. However, nondiabetic *Ccng2^−/−^* mice exhibited significant changes in the extent of pathologic renal injury and fibrosis as compared with WT mice, yet, did not exhibit a significant increase in urinary albumin or the UACR (Figures 2 and 3). These results demonstrate that nondiabetic cyclin G2 deficiency causes tubulointerstitial fibrosis and pathological changes, but is probably not sufficient to cause albuminuria. Together, these data further suggest that cyclin G2 functions as an inhibitory factor for the progression of renal injury and fibrosis.

Recently, emerging evidence has suggested that insulin may be able to attenuate the activation of Wnt signalling via lowering blood levels in renal tissues of diabetic animal models.^54^ Meanwhile, high glucose activated Wnt signalling in renal proximal tubular epithelial cells, whereas inhibition of Wnt by an anti‐LRP6 antibody ameliorated tubulointerstitial fibrosis.^54^ Previously, we reported that cyclin G2 can suppress Wnt/β‐catenin signalling and inhibit gastric cancer cell growth and migration.^45^ Furthermore, cyclin G2 also inhibits the epithelial‐to‐mesenchymal transition by disrupting Wnt/β‐catenin signalling in epithelial ovarian cancer.^55^ In our study, cyclin G2 attenuated Wnt/β‐catenin signalling induced by high‐glucose treatment of HK‐2 cells. In contrast, cyclin G2 deficiency activated Wnt/β‐catenin signalling in our mouse model; this effect was enhanced in mice with STZ‐induced DN. Further experiments demonstrated that the GSK3β inhibitor, CHIR99021, interfered with the inhibitory effects of cyclin G2 on the Wnt signalling pathway as well as on expression of fibrosis‐related proteins (Figure 4). These data provide strong evidence that cyclin G2 acts as a suppressor of Wnt signalling in DN. Binding between Dpr1 and cyclin G2 resulted in Dvl2 degradation via polyubiquitination, which increased the activity of GSK3β, promoted association of β‐catenin with degradation complexes, and interfered with nuclear entry.^45^ Finally, we report here that silencing of Dpr1 interrupted the inhibitory effect of cyclin G2 on Dvl2, β‐catenin and cyclin D1; this indicates that cyclin G2 suppressed Dvl2 expression by binding to Dpr1 and subsequently inhibiting the expression of fibrosis‐related proteins by negatively regulating the Wnt signalling pathway (Figure 5).

CK1 has been shown to phosphorylate Dpr1 and impede the interaction of Dpr1 with Dvl2, which results in the activation of Wnt signalling.^46^ Thus, we demonstrated that cyclin G2 overexpression could influence the binding of CK1 and Dpr1, and inhibit CK1‐mediated phosphorylation of Dpr1. Dpr1 has two known domains: a leucine zipper (LZ) domain at the N‐terminus and the PDZ‐B domain at the C‐terminus.^56^, ^57^ Despite affecting the ability of Dpr1 to inhibit the Wnt signalling pathway, the LZ‐domain is unnecessary for the binding of Dpr1 to Dvl; however, mutation or absence of the PDZ‐B domain does interfere with the binding of Dpr1 and Dvl.^46^ After truncating the Dpr1 protein, we identified an interaction between cyclin G2 and the amino acid domain at residues 1‐619 and 670‐836 of the Dpr1 C‐terminal domain (Figure S4). Prediction analysis showed that Dpr1 Ser762 was a candidate site for CK1‐mediated phosphorylation of Dpr1 (Figure S4). We constructed a Dpr1 Ser762‐mutant vector and found that phosphorylation of Dpr1 and the expression of β‐catenin and cyclin D1 were decreased after cotransfection of the cyclin G2 expression vector and the wild‐type Dpr1 expression vector, while the expression of phosphorylated β‐catenin was increased (Figure 6). These findings have preliminarily identified a new mechanism by which cyclin G2 regulates CK1‐mediated Dpr1 phosphorylation at Ser762 in the C‐terminal PDZ‐B domain.

**Figure 7:**
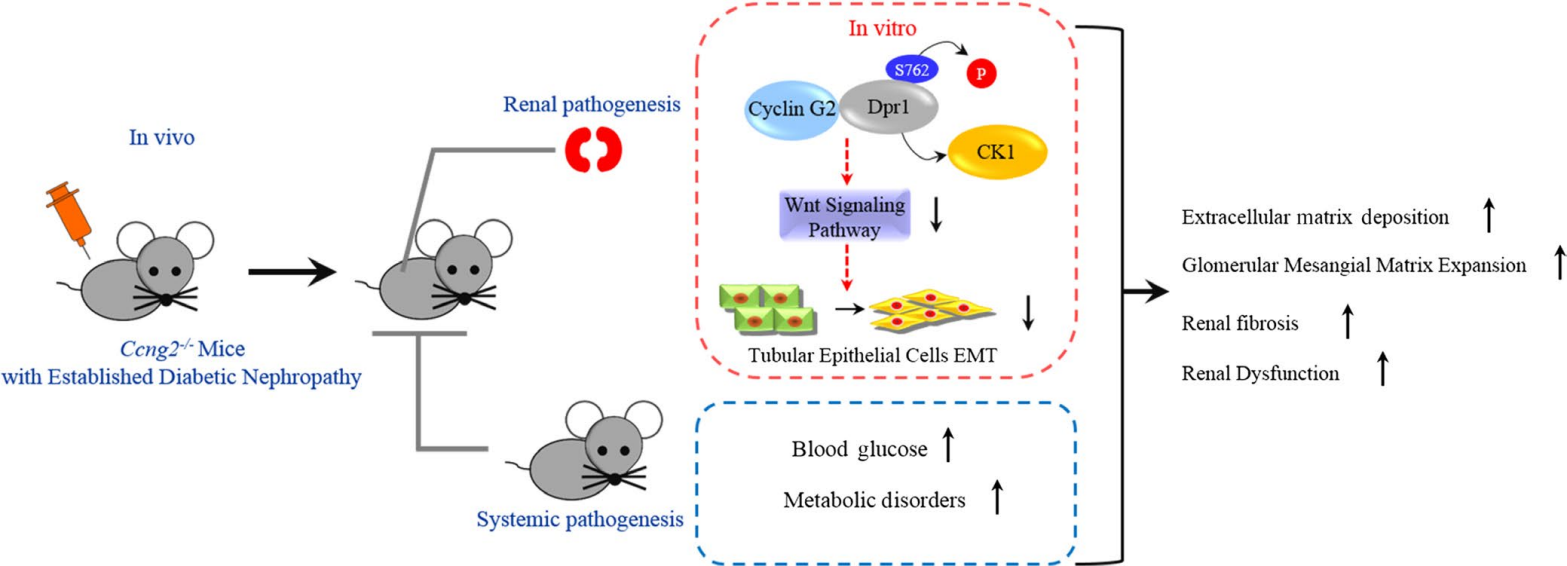
Schematic diagram illustrating the potential effects and underlying mechanisms by which cyclin G2 affects DN. The regulation of the pathological progression by cyclin G2 in the DN mouse kidney proceeds through the repression of Wnt signalling. The interaction of cyclin G2 and Dpr1 affects CK1‐mediated phosphorylation of Dpr1 at Ser762, causes degradation of Dvl2, inhibits the expression of β‐catenin and thereby regulates the activity of the Wnt signalling pathway

In conclusion, our study reveals that cyclin G2 negative regulation of Wnt signalling through Dpr1 is part of a novel mechanism by which tubulointerstitial fibrosis is suppressed in DN. We also demonstrated that the interaction between cyclin G2 and Dpr1 decreased CK1‐mediated phosphorylation of Dpr1 at Ser762, resulting in the degradation of Dvl2 and the inhibition of β‐catenin expression (Figure 7). Our findings delineate a previously unidentified function for cyclin G2, as a protective factor against the pathologic progression of tubulointerstitial fibrosis, and suggest that the interaction between cyclin G2 and the Wnt signalling pathway renders a new therapeutic target for tubulointerstitial fibrosis in DN.

## CONFLICT OF INTEREST

The authors declare that there are no competing interests associated with the manuscript.

## AUTHOR CONTRIBUTIONS

Yang Luo and Qi Liu designed research; Xuesha Xing and Manni sun analysed data; Sen Li, Xiaoyu Hou, Danning Wang and Chenyang Zhao performed animal and cell experiments; Chenyang Zhao wrote the paper; Shusen Wang and Jinlan Gao contributed new reagents or analytic tools.

## Data Availability

All data generated or analysed during this study are included in this published article (and its additional files).
