# Supplementary figures and images for: Cyclin G2 regulates canonical Wnt signalling via interaction with Dapper1 to attenuate tubulointerstitial fibrosis in diabetic nephropathy
Source: J Cell Mol Med. 2020 Jan 24;24(5):2749–60. doi: 10.1111/jcmm.14946 (PMC7077553; doi:10.1111/jcmm.14946)

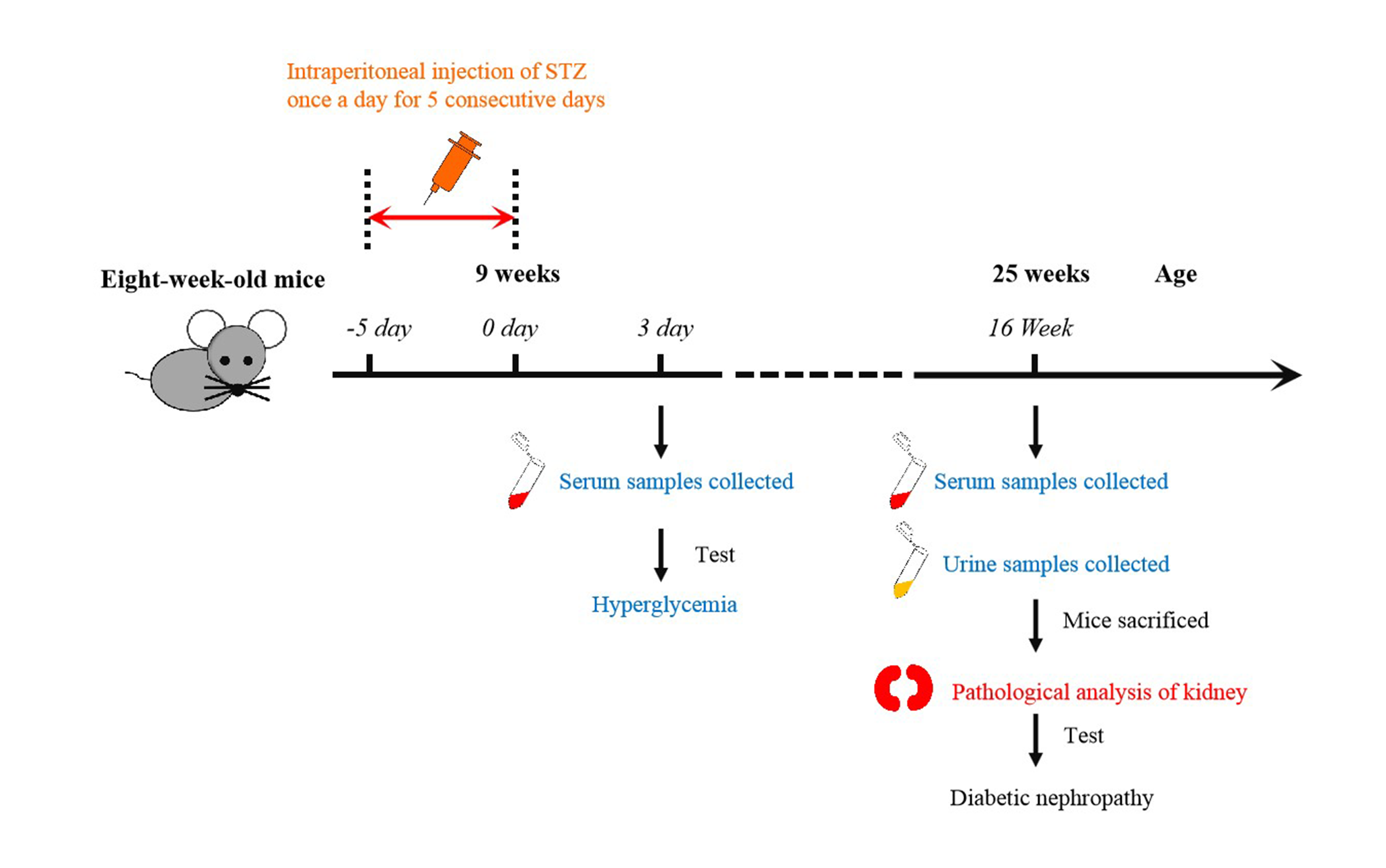

Supplement: Supplementary file 1 [file JCMM-24-2749-s001.png]

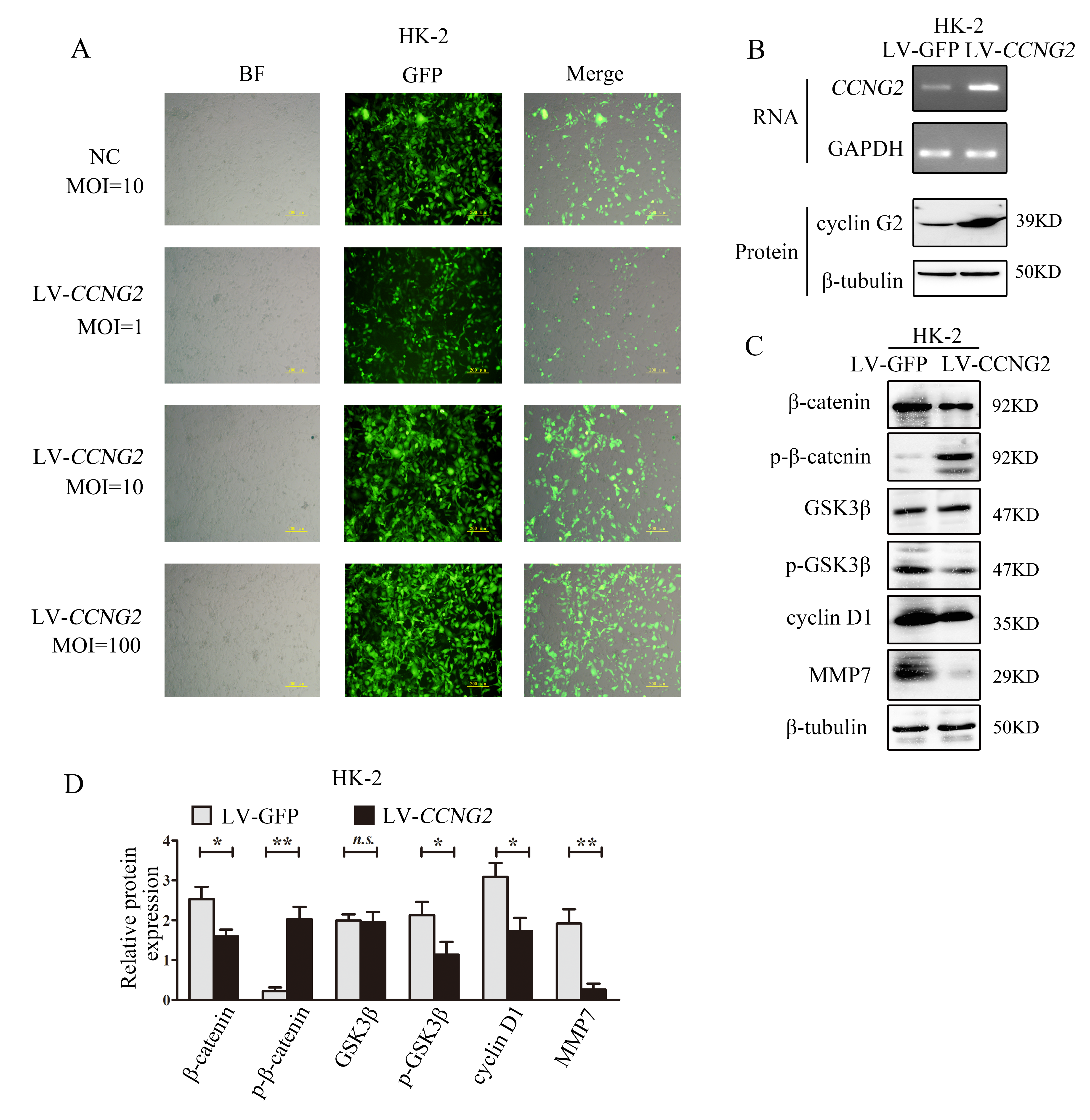

Supplement: Supplementary file 2 [file JCMM-24-2749-s002.png]

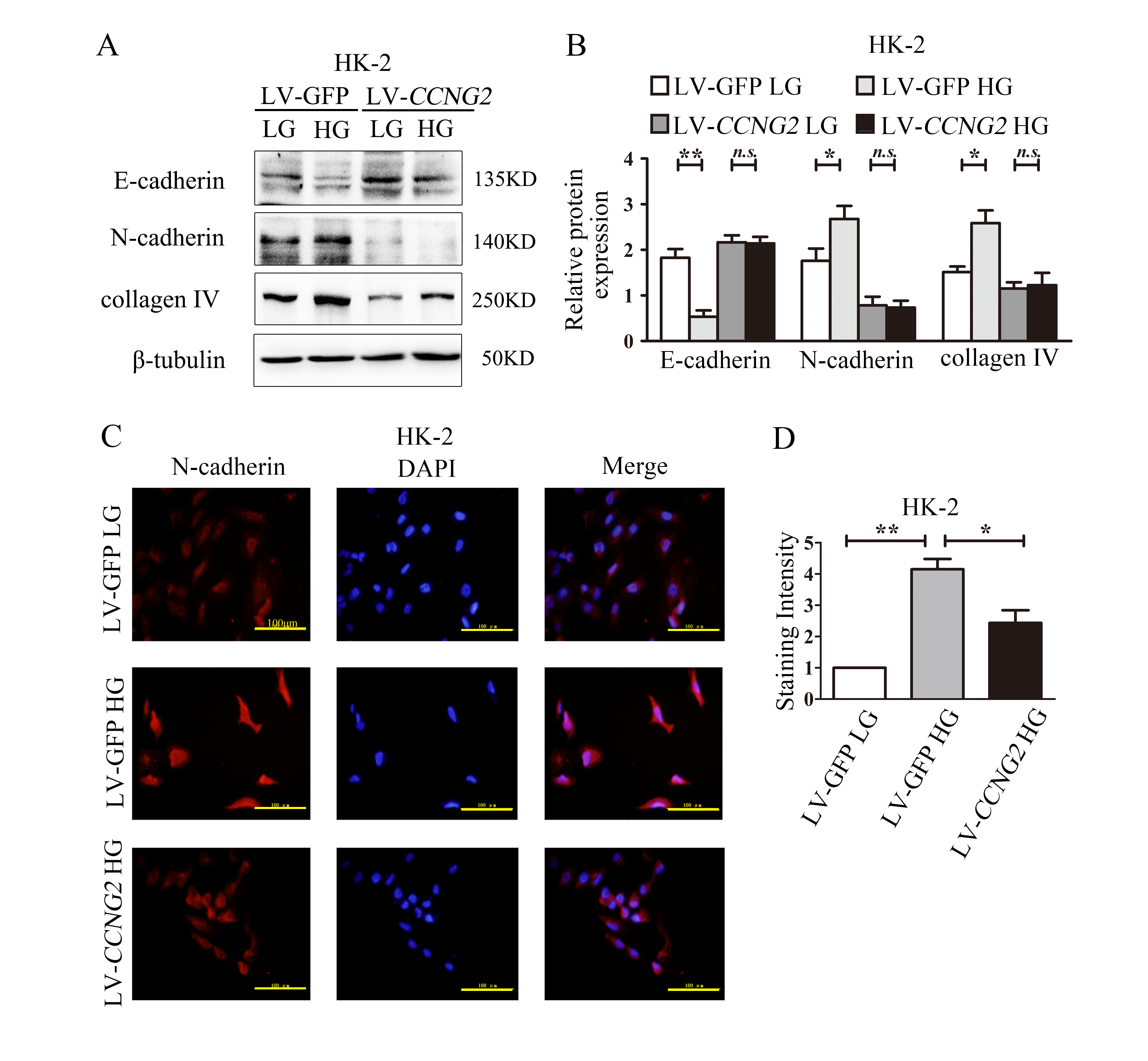

Supplement: Supplementary file 3 [file JCMM-24-2749-s003.png]

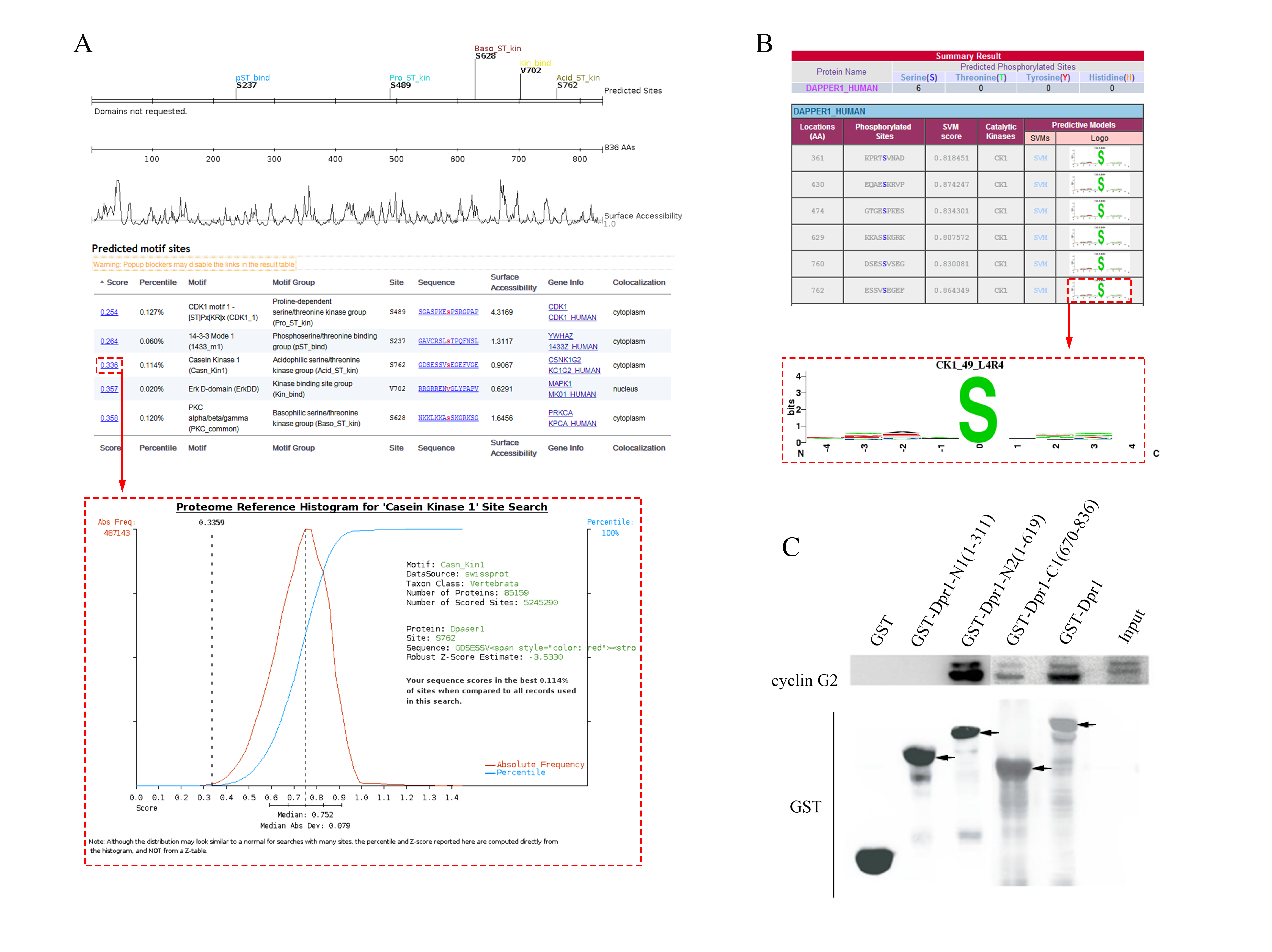

Supplement: Supplementary file 4 [file JCMM-24-2749-s004.png]
